# Abnormal brain diffusivity in participants with persistent neuropsychiatric symptoms after COVID-19

**DOI:** 10.1515/nipt-2022-0016

**Published:** 2023-03-25

**Authors:** Huajun Liang, Thomas Ernst, Kenichi Oishi, Meghann C. Ryan, Edward Herskovits, Eric Cunningham, Eleanor Wilson, Shyamasundaran Kottilil, Linda Chang

**Affiliations:** Diagnostic Radiology and Nuclear Medicine, University of Maryland School of Medicine, Baltimore, MD, USA; Department of Neurology, Johns Hopkins University School of Medicine, Baltimore, MD, USA; Department of Radiology, Johns Hopkins University School of Medicine, Baltimore, MD, USA; Program in Neuroscience, University of Maryland School of Medicine, Baltimore, MD, USA; Department of Medicine, Division of Infectious Disease, Institute of Human Virology, University of Maryland School of Medicine, Baltimore, MD, USA; Department of Neurology, University of Maryland School of Medicine, Baltimore, MD, USA

**Keywords:** amygdala, brain, COVID-19, diffusion tensor imaging, post-COVID conditions

## Abstract

**Objectives:**

We aimed to compare brain white matter integrity in participants with post-COVID-19 conditions (PCC) and healthy controls.

**Methods:**

We compared cognitive performance (NIH Toolbox^®^), psychiatric symptoms and diffusion tensor imaging (DTI) metrics between 23 PCC participants and 24 controls. Fractional anisotropy (FA), axial (AD), radial (RD), and mean (MD) diffusivities were measured in 9 white matter tracts and 6 subcortical regions using MRICloud.

**Results:**

Compared to controls, PCC had similar cognitive performance, but greater psychiatric symptoms and perceived stress, as well as higher FA and lower diffusivities in multiple white matter tracts (ANCOVA-p-values≤0.001–0.048). Amongst women, PCC had higher left amygdala-MD than controls (sex-by-PCC p=0.006). Regardless of COVID-19 history, higher sagittal strata-FA predicted greater fatigue (r=0.48-0.52, p<0.001) in all participants, and higher left amygdala-MD predicted greater fatigue (r=0.61, p<0.001) and anxiety (r=0.69, p<0.001) in women, and higher perceived stress (r=0.45, p=0.002) for all participants.

**Conclusions:**

Microstructural abnormalities are evident in PCC participants averaged six months after COVID-19. The restricted diffusivity (with reduced MD) and higher FA suggest enhanced myelination or increased magnetic susceptibility from iron deposition, as seen in stress conditions. The higher amygdala-MD in female PCC suggests persistent neuroinflammation, which might contribute to their fatigue, anxiety, and perceived stress.

## Introduction

The SARS-CoV-2 virus causes coronavirus disease 2019 (COVID-19), and an estimated one-third of survivors develop neuropsychiatric symptoms within 6 months, known as post-COVID conditions (PCC) [1, 2]. While the prevalence of PCC is higher among patients with a more severe clinical course [1], those with mild acute illness can also develop fatigue, concentration and memory problems (“brain fog”), headaches, anosmia, hypogeusia, anxiety, and/or depression [2].

The exact biological mechanisms underlying these PCC symptoms remain unclear. Evaluation of postmortem brains of patients who died from the acute illness showed activated glia and infiltrated immune cells, suggesting neuroinflammation as a possible biological mechanism [3]. In addition, cerebrospinal fluid (CSF) [4] from hospitalized patients during acute illness contained elevated glial fibrillary acidic protein (GFAP; glial activation marker) and neurofilament light (NfL; axonal damage marker), which also indicate possible brain injury and neuroinflammation during acute infection. However, whether the neuroinflammation persists after recovery from the acute infection is unknown. Recently, an animal study showed that SARS-COV-2 infected rodents with mild respiratory symptoms had persistent microglial activation, subcortical white matter myelin loss, and elevated CSF cytokines and chemokines (e.g. CXCL10, CCL7, CCL2, CCL11) up to 7 weeks post-infection [5], suggesting possible persistent neuroinflammation in PCC.

Neuroinflammation can be non-invasively evaluated in humans using diffusion tensor imaging (DTI). DTI has been widely used to detect brain microstructural abnormalities caused by various neuropathology, including neuroinflammation. DTI evaluates tissue microstructure by measuring the movement of water molecules [6]. Persistent neuroinflammation can cause fiber demyelination, degeneration, and neuronal damage; consequently, water movement becomes more diffuse, corresponding to higher diffusivity indices. DTI studies that evaluated brain abnormalities in COVID-19 found both higher [7–9] and lower [10] white matter diffusivity within six months of acute infection. The discrepancies in the literature may result from differences in the participants’ clinical features. For example, one study reported higher diffusivity in older participants (50–70 years old) who were free from any neurological symptoms during acute infection and at the 3 month follow-up [7]. Conversely, lower diffusivities were also reported in participants who had a high prevalence of neuropsychiatric symptoms during acute infection (68.3%) and 3 months later (55%) [10]. However, no DTI study has specifically evaluated only participants with PCC.

Therefore, we used DTI to compare microstructural integrity in the brain white matter and subcortical grey matter in participants with PCC and those in the control group. We further evaluated whether any abnormalities in DTI metrics were related to the neuropsychiatric symptoms. We hypothesized that (1) recovered COVID-19 participants with PCC would have ongoing neuroinflammation, which would be shown by higher diffusivity and lower fractional anisotropy (FA) in the cerebral white matter compared to those in the controls, and (2) the higher diffusivity and lower FA in the selected regions of interest would predict both poorer emotional health and cognitive performance.

## Participants and methods

### Participants

**Figure 1:**
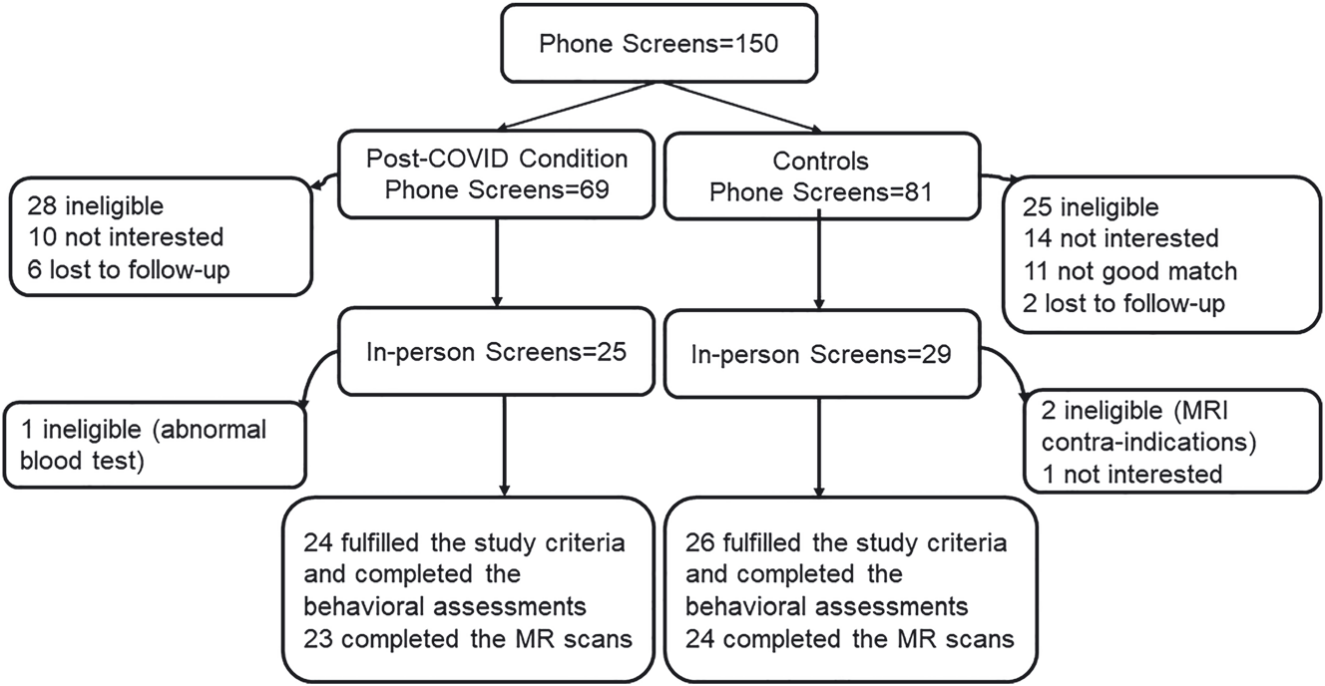
Consort diagram. Showing 28 individuals screened were determined to be ineligible for the post-COVID condition group and 25 determined to be ineligible for the control group. We ultimately studied 24 controls and 26 with PCC who fulfilled all study criteria and completed the assessments.

We enrolled participants with self-reported symptoms consistent with post-COVID condition who also had documented prior COVID-19 (PCC group) and a healthy control group who were likely never infected (see criteria below). Participants were recruited from the local community through website advertisements, flyers, word of mouth, and referrals from healthcare providers and local post-COVID care clinics between February 2021 and February 2022. 150 interested individuals completed the telephone screen. Information on self-reported age, sex, race, and ethnicity of the study subjects were collected during telephone screen. Classifications of self-reported race and ethnicity were used according to the U.S. Census data. Amongst the potentially eligible control participants, those who were matched on age, sex, education level, and race with the participants in the PCC group were invited for further evaluation. Of those who completed telephone screen, 54 (36%) were invited to complete the in-person screening evaluation and provided written informed consent for the study. Four participants were excluded: two had MRI contra-indications, one had significantly elevated plasma levels of alanine transaminase and aspartate transaminase, and one declined to complete the study. Of the remaining 50 participants, three could not complete the MRI scan (two due to excessive shoulder width, one due to a nonremovable metallic pierced object). The final dataset included 24 controls (13 women) and 23 with PCC (15 women) who fulfilled all study criteria and completed both the neuropsychiatric evaluations and brain MRI scans (see Consort Diagram in Figure 1).

Inclusion criteria for both groups were men or women aged 18–75 years who were able to provide informed consent for the study. PCC participants were required to have a documented history of COVID-19 at least 6 weeks earlier and had at least one new cognitive or neuropsychiatric symptom after COVID-19 (i.e., memory complaints, headache, “brain fog”, loss of taste or smell, fatigue, depression or anxiety, sleep disturbances, pain). Control participants were included only if they never had a history of COVID-19 or symptoms related to the illness and had to provide documentation of a negative COVID-19 polymerase chain reaction test within one week prior to enrollment, or a negative COVID-19 rapid antigen test on-site (Abbot BinaxNOW^®^). Participants who received the COVID vaccine were at least 7 days from their last dose to avoid any confounding post-vaccination sequelae. Participants were excluded if they had: (1) a confounding neurological or psychiatric disorder (e.g., stroke, encephalitis from any cause except COVID-19, neurodegenerative disorders, schizophrenia, uncontrolled major depressive/anxiety disorders diagnosed prior to COVID-19, etc.); (2) a severe substance use disorder (as defined by the Diagnostic and Statistical Manual of Mental Disorders 5 criteria) within the past two years except for tobacco use disorders; (3) history of traumatic brain injury with loss of consciousness for >1 h and required hospitalization; (4) any MRI contraindication, or (5) pregnancy or breastfeeding. The study protocols were approved by the Committee on Human Studies at the University of Maryland Baltimore, and a Certificate of Confidentiality was obtained from the National Institute of Health. All participants provided written informed consent.

### Clinical and cognitive assessments

A study physician evaluated all participants during the in-person screening visit to further validate their eligibility, which included a structured evaluation, with a detailed medical history (including review of medical records), physical and neurological examinations, urine toxicology screening, and electrocardiogram. All participants provided either a recent (within one year) complete blood count and comprehensive metabolic panel or completed the screening blood tests on-site. Those with a history of COVID-19 provided their positive test date, any treatments, symptoms during the acute illness, and current post-COVID symptoms and severity. All were assigned a COVID-19 acute illness severity value defined by the 7 Point Endpoint Scale [11]. The Hollingshead Four Factor Index of Socioeconomic Status was used to provide the Index of Social Position (ISP) for all participants [12]. Participants fulfilling all study criteria completed the MRI and the NIH Toolbox^®^ (NIHTB) Cognitive Battery [13], the NIH Toolbox Perceived Stress form (from the Emotion Battery), and selected tests from the Patient-Reported Outcomes Measurement (PROMIS; for the domains of Anxiety, Pain Interference, Fatigue, Psychological Well-Being) [14]. The NIHTB and PROMIS provided fully corrected T scores adjusted for age, sex, race/ethnicity, and education. The data from the NIHTB and PROMIS used for this study is from a subset of the sample published recently [15].

### Image acquisition and processing

All MRI scans were acquired using a 3T Siemens Prisma scanner (Siemens, Erlangen, Germany). A T1-weighted magnetization-prepared rapid gradient-echo (MPRAGE) sequence (TR/TE/TI=2200/4.47/1000 ms, flip angle=12°, FOV=256 mm, 256*256 matrix, thickness=1 mm) and axial fluid-attenuated inversion recovery scan (FLAIR, TR/TE/TI=9100/85/2500 ms, flip angle=150°, FOV 230 mm, 256 × 256 matrix, 3 mm slices) were acquired to examine for possible brain structural abnormalities. All images were reviewed by a Neuroradiologist (**) and none were excluded from this study. DTI was acquired using a spin-echo echo-planar sequence (TR/TE=4200/91 ms, in-plane resolution 2.5 × 2.5 mm, 2.5 mm thickness, four b=0 scans, 30 directions with b=1000 s/mm^2^).

DTI data were processed using the MRICloud (http://www.MRICloud.org) [16], a web-based platform that conducts automated brain MRI quantification, including DTI preprocessing, tensor calculation, and structural parcellation. The multi-atlas label fusion (MALF) method based on the diffeomorphic likelihood fusion algorithm was used to parcellate the target DTI. In the MALF approach, the multiple atlases are transformed into the target image, and the transformed parcels are fused (label fusion) to create the parcellation map specific to the target image [17, 18]. The multi-atlas library used in this study was “Adults_168labels_12atlases_V1” which included the atlases of 12 healthy individuals aged 20–50 years [19]. Fractional anisotropy (FA), mean diffusivity (MD), radial diffusivity (RD), and axial diffusivity (AD) values were obtained for 9 major white matter fiber tracts (corpus callosum, corona radiata, internal capsule, external capsule, cingulum, sagittal stratum, fronto-occipital fasciculus, longitudinal fasciculus, uncinate fasciculus) and MD for 6 subcortical grey matter regions (amygdala, hippocampus, caudate nucleus, putamen, globus pallidus, thalamus).

### Statistical analyses

All data were analyzed using R (version 3.6.3). Demographical data, clinical variables, and NIHTB and PROMIS scores were compared between the two groups using *t*-tests, Chi-square, or Fisher’s exact tests as appropriate. A linear mixed-effects model was used to evaluate the effects of PCC on DTI using PCC status (PCC or control) and PCC status-by-sex as effects of interest, hemisphere and sub-region as repeated factors, subject ID as a random factor, and age and sex as covariates. The model was repeated without the PCC status-by-sex interaction term if insignificant. p-values<0.05 were considered significant.

Separate models were created to examine FA and MD values in the corpus callosum (CC, genu, body, and splenium), corona radiata (CR, anterior, posterior, and superior), internal capsule (IC, anterior limb, posterior limb, and retrolenticular of IC), external capsule, cingulum (cingulum cingulate or CGC, cingulum hippocampus), sagittal stratum (SS), fronto-occipital fasciculus (superior and inferior fronto-occipital fasciculus), superior longitudinal fasciculus (SLF), and uncinate fasciculus. Due to the crossing fibers, only MD was evaluated in the basal ganglia (BG, caudate nucleus, putamen, globus pallidus), thalamus, amygdala, and hippocampus.

Post-hoc analyses were performed for AD and RD when PCC status or PCC-by-sex had at least a trend-level (p<0.1) effects on MD. Similarly, post-hoc analyses were performed for sub-regions when there was a significant/trend level group difference on effects of interest in the main region. For DTI measures with significant effects of interest, a linear model evaluated their associations with (1) the time since COVID-19 diagnosis, and (2) cognitive performance and emotional health measures that showed group differences.

## Results

### Participant characteristics and PCC symptoms

Participants with PCC and controls were similar in age, proportions of sex, race, education level, and ISP (Table 1). The Race/ethnicity distribution of our participation were: Asian (4% of total participants, two in the control group), Black (38% of total participants, eight in PCC group, 10 in the control group), Hispanic (6% of total participants, three in the control group), Two or more races (2% of total participants, one in PCC group), and White (49% of total participants, 14 in PCC group, 9 in the control group). The PCC group tended to have a higher body mass index (+13.4%, p=0.056) than the controls. The two groups had similar percentages of individuals with hypertension or diabetes prior to COVID-19, as well as the presence of white matter hyperintensities on the MRI scans, indicating a similar level of vascular disease burden. The PCC group tended to have fewer participants who were vaccinated for COVID-19 than the control group but the group difference was not significant (p=0.051). PCC participants had developed natural immunity, which may explain their nonsignificantly lower rate of vaccination compared to those who were likely never infected. The two groups also had similar prevalence for depression or anxiety disorders prior to COVID-19, as well as lifetime or current tobacco, marijuana, or alcohol use.

**Table 1:** Participants characteristics.

	Control, n=24	PCC, n=23	p-Value
Age, years	44.3 ± 12.5	44.1 ± 12.2	0.726^a^
Sex (#woman, %)	13 (54.2%)	15 (65.2%)	0.635^b^
Race (#white/#non-white^f^)	9/15	14/9	0.190^b^
Index of socioeconomic position (mean ± SD)	29.3 ± 14.8	30.3 ± 14.7	0.208^a^
Education level (#, %)			0.134^b^
High school or below	6 (25%)	11 (47.8%)	
College	8 (33.3%)	8 (24.8%)	
Above college	10 (41.7%)	4 (17.4%)	
Vaccinated for COVID-19^d^ (#, %)	19 (79.2%)	12 (52.2%)	0.051^b^
Vascular disease risk factors			
Body Mass index (mean ± SD)	27.7 ± 6.6	32.0 ± 8.3	0.056^a^
Hypertension (#, %)	1 (4.2%)	6 (26.1%)	0.09^b^
Diabetes (#, %)	0	4 (17.4%)	0.107^c^
White matter lesion(s) (#, %)	15 (62.5%)	15 (65.2%)	0.846^b^
Depression or anxiety disorders prior to COVID-19 (#, %)			
Depression^e^	3 (12.5%)	5 (21.7%)	0.400^b^
Anxiety^e^	2 (8.3%)	6 (26.1%)	0.106^b^
History of Substance use (#, %)			
Lifetime tobacco use	9 (37.5%)	9 (39.1%)	1^b^
Past month tobacco use	1 (4.2%)	1 (4.3%)	1^c^
Lifetime marijuana use	6 (25%)	10 (43.5%)	0.304^b^
Past month marijuana use	1 (4.2%)	1 (4.3%)	1^c^
Lifetime alcohol use	20 (83.3%)	19 (82.6%)	1^b^
Past month alcohol use	17 (70.8%)	15 (60%)	0.920^b^
Self-reported symptoms (n, %)			
Concentration problems		21 (87.5)	
Fatigue		20 (83.3)	
Memory problems		19 (79.2)	
Depression or anxiety		16 (66.7)	
Confusion		17 (70.8)	
Myalgia		14 (58.5)	
Headaches		13 (54.2)	
Gait disturbances		13 (54.2)	
Hyposmia		12 (50)	
Sleep disturbances		12 (50)	
Dysgeusia		11 (45.8)	
Paresthesia		11 (45.8)	
Dizziness		10 (41.7)	
Visual disturbances		9 (37.5)	
Lightheadedness		9 (37.5)	
Coordination problems		9 (37.5)	
Urinary problems		5(20.8)	
Postural instability		4 (16.7)	
Other neurological^f^		6 (25.0)	

^a^T test, ^b^Chi-Square test, ^c^Fisher’s exact test; ISP, index of socioeconomic position; PCC, participants with post-COVID conditions. ^d^COVID-19 vaccine status was unknown for three participants in the control group, ^e^two in each group were on medications for depression; one in control and two in PCC were on medication for anxiety. ^f^Other neurological symptoms included: hand tremors (n=3) and tinnitus (n=3, 1 with hearing reduction). Race/ethnicity of Non-White included: Black (8 in PCC group, 10 in the control group), Asian (2 in the control group), Hispanic (3 in the control group), and Two or more races (1 in PCC group).

The median time since COVID-19 diagnosis was 182 days (range: 42–484 days). Among the PCC participants, eight were hospitalized (6 men). The most reported neuropsychiatric symptoms were concentration problems (87%), fatigue (83%), and memory problems (79%) (Table 1).

PCC participants and controls had similar performance on all cognitive assessments. However, the PCC group had higher levels of depression (p=0.001), anxiety (p=0.001), fatigue (p<0.001), Pain-Interference (p<0.001), perceived stress (p=0.001), and poorer global mental health (p<0.001) compared to those in the controls (Table 2).

**Table 2:** NIHToolbox cognitive battery, perceived stress, and PROMIS health evaluation T scores.

Domain	Assessment	Controls n=23	PCC n=24	t-test p-Value
Attention/Executive function	Flanker inhibitory control and attention test	46.92 ± 11.13	44.96 ± 10.14	0.532
Episodic memory	Picture sequence memory test	53.67 ± 9.37	52.35 ± 10.72	0.655
Working memory	List sorting working memory test	49.08 ± 9.97	51.52 ± 8.29	0.368
Language	Picture vocabulary test	52.25 ± 13.67	51.22 ± 9.57	0.767
	Oral reading recognition test	54.83 ± 7.09	52.48 ± 8.15	0.296
Executive function	Dimensional change card sort test	50.25 ± 13.76	52.70 ± 12.85	0.532
Processing speed	Pattern comparison processing speed test	54.42 ± 16.32	55.26 ± 12.85	0.845
	Oral symbol digit test^a^	84.08 ± 17.40	91.39 ± 17.59	0.600
Immediate recall	Auditory verbal learning test (rey)^a^	25.21 ± 7.48	25.39 ± 4.76	0.921
Composite scores	Fluid cognition	51.04 ± 12.97	51.96 ± 10.59	0.793
	Crystallized cognition	53.75 ± 10.21	52.45 ± 9.00	0.651
	Total cognition	52.79 ± 9.57	52.86 ± 9.38	0.980
Self-report mental and physical health	Depression	45.25 ± 6.86	53.27 ± 9.11	0.001
	Anxiety	46.74 ± 8.65	56.55 ± 10.45	0.001
	Fatigue	39.88 ± 8.09	56.71 ± 9.73	<0.001
	Pain interference	41.71 ± 6.1	55.93 ± 9.27	<0.001
	Pain intensity	34.21 ± 5.92	46.20 ± 9.27	<0.001
	Pain quality	32.81 ± 5.84	46.20 ± 8.45	<0.001
	Pain behavior	41.10 ± 8.52	55.19 ± 8.85	<0.001
	Global mental health	53.57 ± 8.61	41.62 ± 9.44	<0.001
	Global score health items	57.40 ± 8.22	36.8 ± 6.92	<0.001
	Perceived stress	46.04 ± 11.56	57.74 ± 11.28	0.001

^a^Raw score. PCC, participant group with the post-COVID conditions.

Of the 47 scans reviewed, nine (six PCC and three controls) were determined to be abnormal. Of these 9 scans, four had more than age-related white matter lesions (3 in PCC group), two had lacunar infarcts (both in PCC group), one had some microhemorrhages (PCC group), one had both a small (6 mm) old and silent infarct and a small microhemorrhage (control group), and one had greater than age-related central atrophy (control group). None of these 9 participants had extreme values on their DTI measures (defined as more than two standard deviations from average); therefore, all DTI scans were included in this study.

### Diffusion findings in PCC

**Figure 2:**
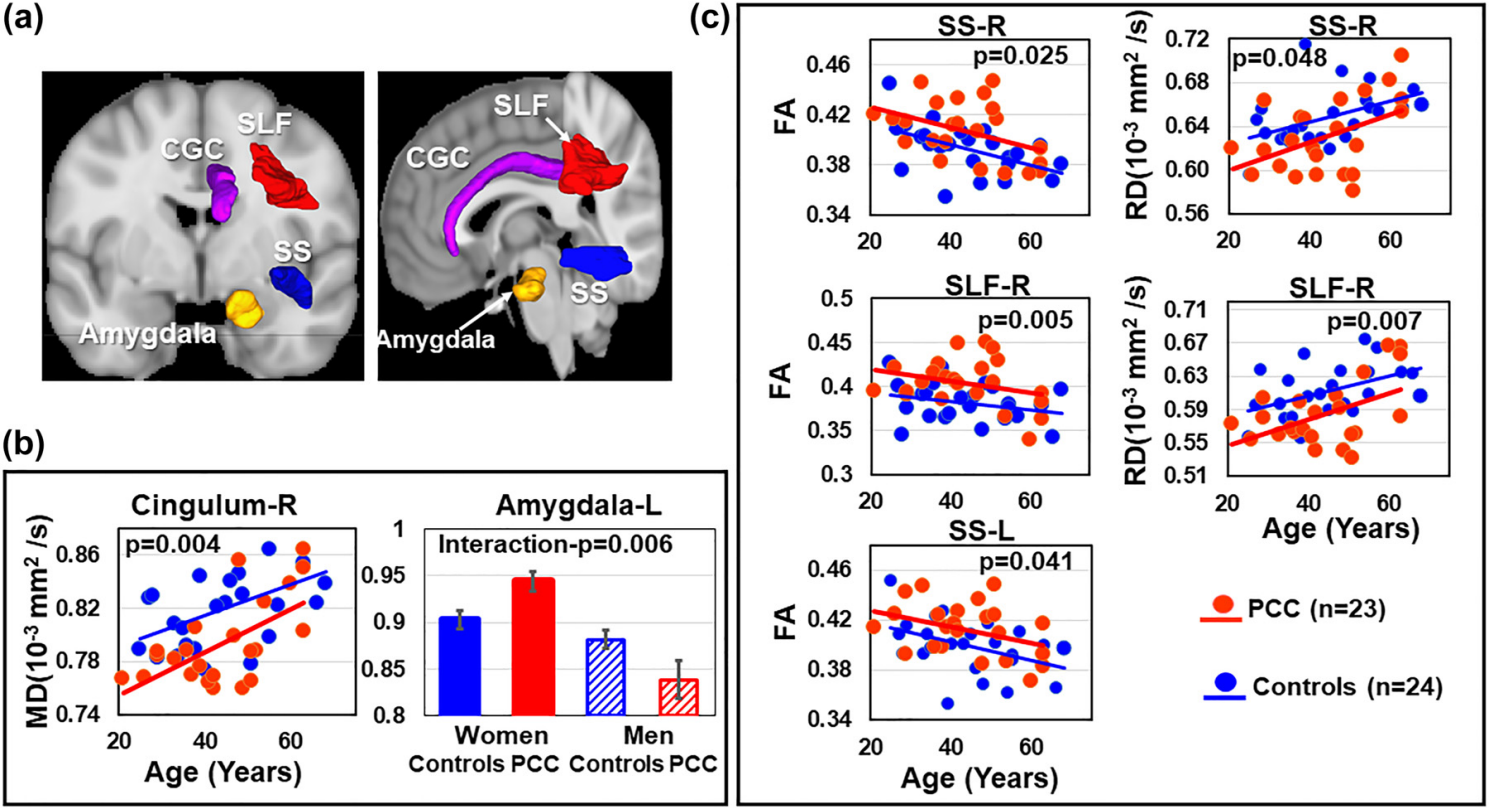
Participants with PCC had higher FA and lower MD than control participants. (a) Brain regions showing significant group differences. (b) Participants with PCC had lower MD than controls in the right cingulum (CGC). Women with PCC had a trend of higher MD in the left amygdala than women controls (0.944 ± 0.05 vs. 0.903 ± 0.05, post hoc p=0.038), but men with PCC showed similar MD to men controls (PCC: 0.839 ± 0.04 vs. controls: 0.882 ± 0.05, post hoc p=0.065). (c) Participants with PCC had higher FA and lower RD in the right SS and right superior longitudinal fasciculus (SLF), and higher FA than controls in left sagittal strata (SS). PCC, participants with post-COVID conditions; FA, fractional anisotropy; MD, mean diffusivity; RD, radial diffusivity; R, Right side; L, Left side.

Compared to controls, PCC participants had lower MD in the CGC (CGC-p=0.008, right CGC-p=0.004, Figure 2b and Table 3). However, the left amygdala-MD had a PCC status-by-sex interaction (interaction-p=0.006). Specifically, in the left amygdala, women with PCC had higher MD than control women (0.94 ± 0.05 vs. 0.90 ± 0.05, *post hoc* p=0.038), whereas the men in both groups had similar MD (PCC: 0.840 ± 0.04 vs. controls: 0.885 ± 0.05, *post hoc* p=0.065, Figure 2b and Table 3). In addition, PCC participants had higher FA bilaterally in the SS (SS-p=0.021; left SS-p=0.041; right SS-p=0.025) and SLF (SLF-p=0.017, right SLF-p=0.005) (Figure 2c and Table 3). These same regions also showed lower RD in PCC compared to controls (right SS-p=0.048, right SLF-p=0.007).

**Table 3:** DTI measures in brain regions of interest (ROIs) with PCC effects.

ROIs	Linear Mixed Model p-Values					
	Age	Sex	Hemisphere	Subregion	Group	Sex-by-Group
Fractional Anisotropy						
Corpus callosum	0.011	0.894	0.164	<0.001	0.207	N/A
Corona radiata	0.005	0.804	0.228	<0.001	0.204	N/A
Internal capsule	0.199	0.258	0.261	<0.001	0.419	N/A
External capsule	0.114	0.241	<0.001	N/A	0.323	N/A
Cingulum	0.265	0.475	<0.001	<0.001	0.816	N/A
Sagittal stratum	0.007	0.088	<0.001	N/A	0.021	N/A
Sagittal stratum left^a^	*0.022*	*0.566*	*N/A*	*N/A*	*0.041*	N/A
Sagittal stratum right^a^	*0.009*	*0.956*	*N/A*	*N/A*	*0.025*	N/A
Superior longitudinal fasciculus	0.086	0.403	0.388	N/A	0.017	N/A
Superior longitudinal fasciculus left^a^	*0.101*	*0.426*	*N/A*	*N/A*	*0.132*	N/A
Superior longitudinal fasciculus right^a^	*0.108*	*0.524*	*N/A*	*N/A*	*0.005*	N/A
Fronto-occipital fasciculus	0.069	0.925	0.073	0.126	0.582	N/A
Uncinate fasciculus	0.359	0.496	0.004	N/A	0.347	N/A
Mean diffusivity, mm^2^/s × 1000						
Corpus callosum	0.009	0.023	<0.001	<0.001	0.124	N/A
Corona radiata	0.004	0.047	<0.001	<0.001	0.291	N/A
Internal capsule	0.088	0.123	<0.001	<0.001	0.419	N/A
Cingulum	0.001	0.279	<0.001	<0.001	0.073	N/A
Cingulum cingulate	*<0.001*	*0.077*	*<0.001*	*N/A*	*0.008*	*N/A*
Left^a^	*0.007*	*0.249*	*N/A*	*N/A*	*0.076*	*N/A*
Right^a^	*0.001*	*0.043*	*N/A*	*N/A*	*0.004*	*N/A*
Cingulum hippocampus	*0.028*	*0.813*	*0.354*	*N/A*	*0.413*	*N/A*
Sagittal stratum	0.026	0.107	0.011	N/A	0.384	N/A
Superior longitudinal fasciculus	0.002	0.078	<0.001	N/A	0.210	N/A
Fronto-occipital fasciculus	0.097	0.088	0.043	<0.001	0.824	N/A
Uncinate fasciculus	0.374	0.189	0.130	N/A	0.320	N/A
Basal ganglia	0.002	0.512	0.741	<0.001	0.801	N/A
Amygdala	0.023	0.053	<0.001	N/A	0.405	0.082
Left^a^	*0.030*	*0.292*	*N/A*	*N/A*	*0.031*	*0.006*
Right^a^	*0.078*	*0.026*	*N/A*	*N/A*	*0.509*	*0.793*
Hippocampus	0.809	0.774	0.001	N/A	0.605	N/A
Thalamus	0.007	0.595	<0.001	N/A	0.956	N/A
Nucleus accumbens	0.352	0.221	<0.001	N/A	0.335	N/A
Radial diffusivity (mm^2^/s × 1000)						
Sagittal stratum left^a^	*0.037*	*0.240*	*N/A*	*N/A*	*0.290*	*N/A*
Sagittal stratum right^a^	*0.010*	*0.021*	*N/A*	*N/A*	*0.048*	*N/A*
Superior longitudinal fasciculus right^a^	*0.004*	*0.054*	*N/A*	*N/A*	*0.007*	*N/A*
Axial diffusivity (mm^2^/s × 1000)						
Sagittal stratum left^a^	*0.787*	*0.984*	*N/A*	*N/A*	*0.065*	*N/A*
Sagittal stratum right^a^	*0.208*	*0.639*	*N/A*	*N/A*	*0.979*	*N/A*
Superior longitudinal fasciculus right^a^	*0.006*	*0.019*	*N/A*	*N/A*	*0.844*	*N/A*

^a^ANCOVA, N/A, factor was not included in the final model. Corpus callosum includes bilateral genu, body, and splenium of the corpus callosum; Corona radiata includes bilateral anterior, posterior, and superior corona radiata; Internal capsule includes anterior limb, posterior limb, and retrolenticular internal capsule, Cingulum includes bilateral cingulum cingulate and cingulum hippocampus; Fronto-occipital fasciculus includes bilateral superior and inferior fronto-occipital fasciculus; Basal ganglia include bilateral caudate, putamen, and globus pallidus. PCC, participant group with the post-COVID conditions. Italic values indicate those from post-hoc analyses.

### DTI measures predict neuropsychiatric symptoms

**Figure 3:**
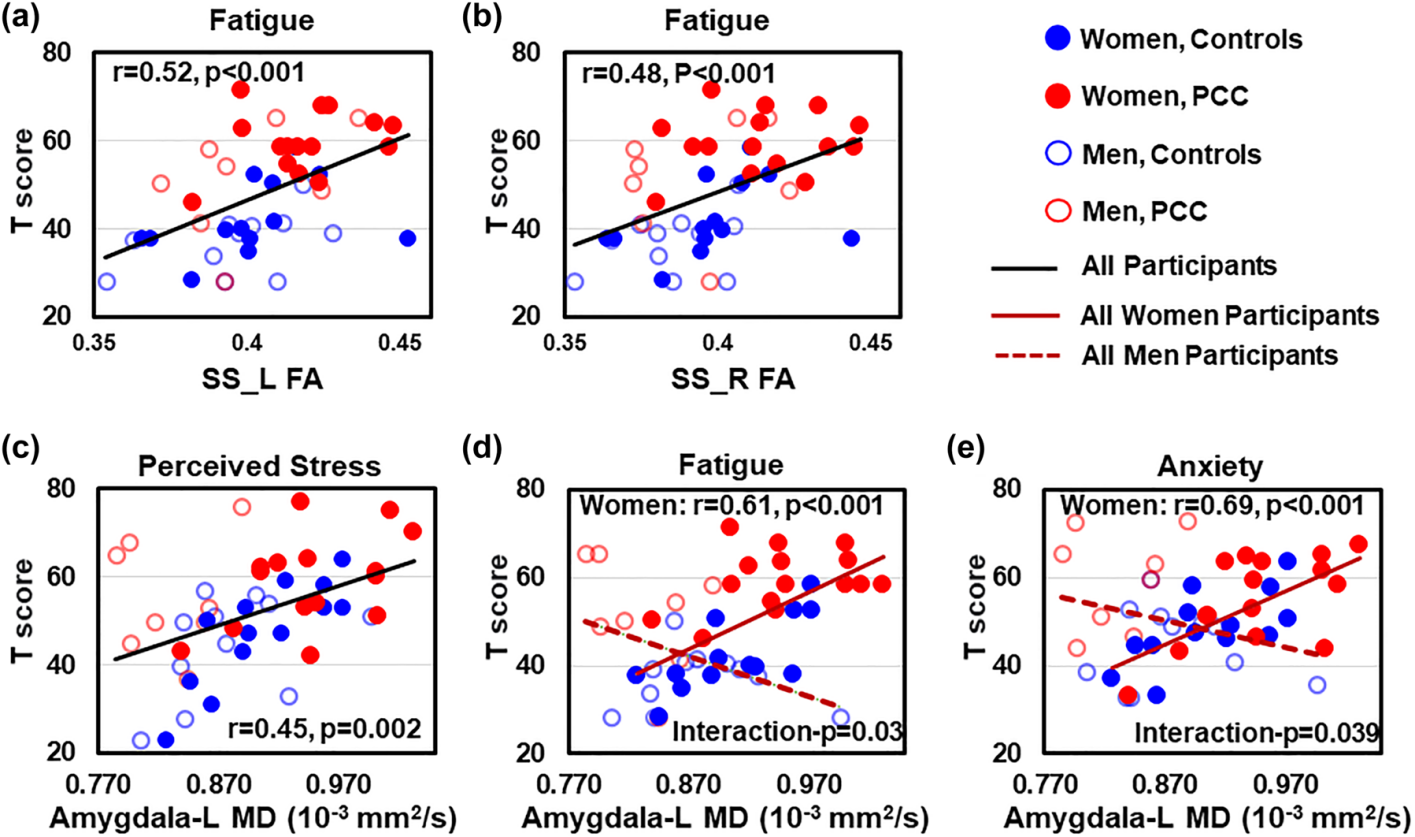
DTI measures predict psychological function. Across all participants, greater fatigue is predicted by higher FA in the left (r=0.52, p<0.001); (a) and right (r=0.48, p<0.001); (b) sagittal strata (SS). (c) Higher MD in the left amygdala predicts greater perceived stress in all participants (r=0.45, p=0.002), regardless of COVID status. (d and e) In women but not in men, higher MD in the left amygdala predicts greater fatigue (r=0.61, p<0.001, interaction-p=0.03) and anxiety (r=0.69, p<0.001, interaction-p=0.039), regardless of COVID status. PCC, participants with post-COVID conditions.

Across all participants, higher FA in the bilateral SS predicted higher fatigue T-scores (left: r=0.52, p<0.001; right: r=0.48, p<0.001) (Figure 3a–b), and higher MD in the left amygdala predicted greater perceived stress (r=0.45, p=0.002, Figure 3c). Regardless of PCC status, women with higher MD in the left amygdala endorsed greater symptoms of fatigue (r=0.61, p<0.001, PCC-by-sex interaction-p=0.03) and anxiety (r=0.69, p<0.001, PCC-by-sex interaction-p=0.039, Figure 3d–e) compared to men. However, none of the DTI measures correlated with time since COVID-19 diagnosis.

## Discussion

This study has the following main findings: (1) Compared to non-infected controls, participants with PCC had higher FA and lower diffusivities in six of the nine white matter tracts indicating more restricted diffusivities in these brain regions; (2) across all participants, higher FA bilaterally in the SS was related to greater fatigue; (3) MD in the left amygdala was sex-specific, higher in women with PCC but not in men with PCC relative to the sex-matched controls; and (4) higher left amygdala MD predicted greater fatigue and anxiety in women and higher perceived stress for all participants.

### Greater stress and emotional distress but normal cognitive function in PCC

To date, studies of participants with PCC rarely used objective assessments to evaluate multiple cognitive domains to assess the neuropsychiatric complaints. Although more than 80% of our PCC participants reported memory or concentration problems, they had relatively normal cognitive performances compared to the controls, as reported in our larger cohort [15]. These findings are consistent with a previous study that evaluated 31 participants with PCC who complained about brain fog but performed normally on a 51-min cognitive assessment [20]. Furthermore, our participants with PCC endorsed greater perceived stress, fatigue, pain, and overall poorer emotional health compared to controls, which is consistent with our hypothesis and with previous studies [1, 2].

### Abnormal white matter diffusivities in PCC

Our finding of white matter restricted diffusivity with lower MD and higher FA in PCC relative to control groups is consistent with a prior DTI study [10] but opposite from our initial expectation of neuroinflammation-induced elevated white matter MD. Our finding is consistent with two recent studies that examined the CSF of nearly 150 patients with PCC, which found that none of the CSF samples contained SARS-CoV-2 RNA, intrathecal SARS-CoV-2 antibodies, or inflammatory markers, suggesting no on-going neuroinflammation in PCC patients [21, 22]. However, our findings differ from several DTI studies that reported higher diffusivities and lower FA in post-COVID patients (with or without PCC) compared to controls [7–9]. These prior findings of high diffusivities in recovered COVID patients are consistent with postmortem [3] studies that reporting acute neuroinflammation and neuronal injury, and in animal studies [5] demonstrating persistent neuroinflammation. However, prior DTI studies did not focus on participants with PCC. Therefore, the abnormally greater diffusivities found in the prior COVID studies may reflect more acute or subacute illness rather than PCC.

In our PCC participants, the lower than normal MD reflects restricted diffusivity in the white matter tracts and indicates more hindrance of water movement (intra-axonal, extra-axonal, or extra-cellular compartments); the higher than normal FA values represent more coherent or compact fibers [6]. Several physical or pathological processes in the brain that can lead to lower diffusivity and higher FA include enhanced myelination [6], increased magnetic susceptibilities [23], and cytotoxic edema [6]. Enhanced myelination, such as that seen during normal neurodevelopment or a repair process in our PCC participants, would lead to more compact fibers, increasing water movement parallel to the axons while reducing water movements perpendicular to the fibers [6].

Another condition that can lead to enhanced myelination and fiber reorganization, represented restricted diffusion and elevated FA, is chronic stress [24–27]. First, stress activates neurons and the release of glucocorticoids, which can trigger oligodendrogenesis to enhance myelination [24]. Patients with post-traumatic stress disorder (PTSD) for 7 years showed higher myelin content, assessed via myelin water imaging, which indirectly measures water diffusivities between neighboring myelin layers, in the cingulum, corpus callosum, and internal capsule compared to non-PTSD controls [25]. Low diffusivity and high FA were also observed in individuals exposed to traumatic events but did not have PTSD [26, 27]. Second, stress and related symptoms can be predicted by elevated FA or lowered diffusivity. Perceived COVID-19 stress is often accompanied by fatigue, depression, and anxiety symptoms [28]; all of these symptoms were observed in our PCC participants. In addition, we observed that PCC-related fatigue was predicted by higher FA in the SS, which is consistent with some prior DTI studies that found higher FA and lower diffusivity in the BCC, PTR, and SLF, that correlated with greater persistent distress, anxiety, and depression in post-COVID participants [8, 29]. Similarly, in non-PTSD trauma survivors, persistently elevated FA in the cingulate predicted higher stress 1 year later [30] and poorer remission in PTSD patients [30, 31]. Therefore, future studies of individuals with PCC need to investigate the role of the stress response and whether white matter diffusivity normalizes with resolution of stress and neuropsychiatric symptoms.

The higher FA and lower diffusivities in our PCC participants may also be due to increased magnetic susceptibility from iron deposition [23]. With neuroinflammation, activated microglia and astrocytes express higher levels of hepcidin, which might contribute to iron accumulation in neurons and microglia [32]. Iron dysmetabolism was observed in patients with COVID-19; those with more severe infection had higher serum levels of hepcidin and ferritin compared to those with milder COVID-19 [33]. Iron overload may lead to oxidative stress and antioxidant deficiency, with lower plasma glutathione (GSH) in patients with severe COVID-19 [34]. Our PCC patients, in fact, had lower than normal brain GSH levels in the frontal gray matter compared to controls [35]. In another study, lower GSH in the anterior cingulate cortex was also associated with more depressive symptoms 4 months after COVID-19 [36]. Lastly, cytotoxic edema, which is typically observed during the early days of an acute stroke, leads to axonal swelling, reduced membrane permeability and lowered diffusivity on diffusion-weighted MRI. However, cytotoxic edema is typically not associated with higher levels of FA [6] and thus an unlikely explanation for our findings.

### Sex-specific microstructural changes in the amygdala in PCC

MD in the left amygdala was higher than normal in women with PCC, but not in men with PCC. The higher MD in amygdala may occur during amygdala over-activation (e.g., by stress), increased blood flow, and/or microglial activation with ongoing neuroinflammation [37]. This sex-specific abnormality is consistent with prior findings that women, compared to men, have greater amygdala responses (increased blood flow) during psychosocial stress-activated functional MRI [38]. Furthermore, we found higher amygdala-MD predicted greater perceived stress, anxiety, and fatigue – independent of COVID-19 history. These findings support the well-known role of the amygdala in stress perception and recent studies evaluating pandemic stress on the general population [37, 39, 40]. Our finding of elevated MD in women with PCC is also consistent with a recent study that found enlarged bilateral amygdala in uninfected healthy individuals during the pandemic lockdown, but the amygdala volumes normalized gradually after the lockdown was lifted [39].

### Limitations and future studies

This study has several limitations. First, due to the cross-sectional design, we cannot definitively attribute the brain microstructural abnormalities directly to symptoms related to PCC. Second, we could not ascertain that none of our control participants had a prior asymptomatic SARS-CoV2 infection, since the serologic antibody tests for past infection were unavailable or less accessible during this project period. However, even the current serologic tests are only able to identify asymptomatic infection that occurred within the past 6–10 months [41]. Our control participants never had any COVID-related symptoms and were required to provide a recent negative PCR test for COVID-19, or had a negative antigen test on-site during the visit. In addition, asymptomatic infection rate was only about 8.4% during the Delta wave in general population and would be even less prevalent amongst adults [42]. Therefore, the likelihood that we might have enrolled a control participant with prior asymptomatic infection is very low. Third, although the PCC group tended to have higher average BMI and more participants with comorbidities (i.e., hypertension, diabetes) compared to controls, these conditions typically correlated with lower FA or higher diffusivity [43–45], which are opposite from the findings in our study. Therefore, our DTI results could not be confounded by higher BMI, hypertension or diabetes in the PCC group. Future studies should include antibody tests for all control subjects, longitudinal follow-ups in those with PCC, and a COVID-19 recovered group without PCC to further delineate the impact of acute or subacute COVID-19 versus PCC.

## Conclusions

Compared to the controls, recovered COVID participants with persistent neuropsychiatric symptoms had higher FA and restricted diffusivity in several white matter regions and higher mean diffusivity in the left amygdala. These microstructural abnormalities in the white matter were contrary to what we initially hypothesized. These findings may reflect enhanced myelination resulting from stress, increased magnetic susceptibility from iron dysmetabolism, or less likely, ongoing cytotoxic edema. Importantly, our findings demonstrate that brain abnormalities persist for an average of six months or longer in patients with long COVID symptoms. Longitudinal studies are needed to evaluate whether these brain microstructural abnormalities will normalize along with the resolution of PCC related symptoms.
